# Shallow Silicon Vacancy Centers with Lifetime-Limited Optical Linewidths in Diamond Nanostructures

**DOI:** 10.1021/acs.nanolett.3c03145

**Published:** 2023-11-21

**Authors:** Josh A. Zuber, Minghao Li, Marcel.li Grimau Puigibert, Jodok Happacher, Patrick Reiser, Brendan J. Shields, Patrick Maletinsky

**Affiliations:** † Department of Physics, University of Basel, CH-4056 Basel, Switzerland; ‡ Swiss Nanoscience Institute, University of Basel, CH-4056 Basel, Switzerland

**Keywords:** silicon vacancy center, diamond nanostructures, optical coherence, charge-stabilization, quantum sensing

## Abstract

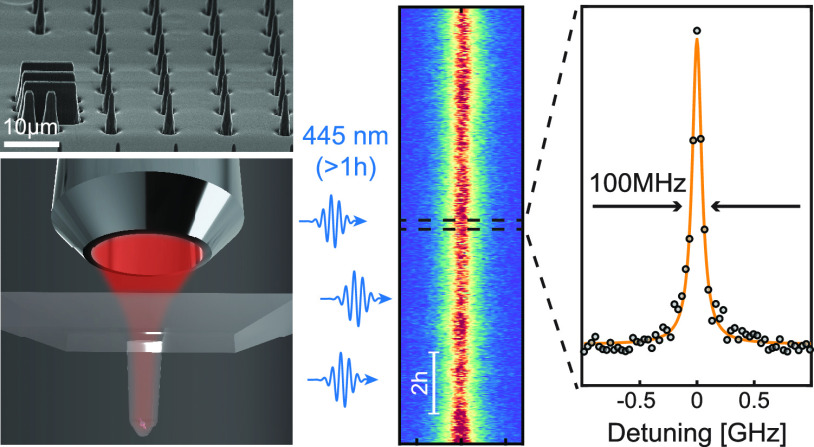

The negatively charged silicon vacancy center (SiV^–^) in diamond is a promising, yet underexplored candidate for single-spin quantum sensing at sub-kelvin temperatures and tesla-range magnetic fields. A key ingredient for such applications is the ability to perform all-optical, coherent addressing of the electronic spin of near-surface SiV^–^ centers. We present a robust and scalable approach for creating individual, ∼50 nm deep SiV^–^ with lifetime-limited optical linewidths in diamond nanopillars through an easy-to-realize and persistent optical charge-stabilization scheme. The latter is based on single, prolonged 445 nm laser illumination that enables continuous photoluminescence excitation spectroscopy without the need for any further charge stabilization or repumping. Our results constitute a key step toward the use of near-surface, optically coherent SiV^–^ for sensing under extreme conditions, and offer a powerful approach for stabilizing the charge-environment of diamond color centers for quantum technology applications.

Diamond color centers represent the backbone for many research directions in quantum technologies, including sensing,^1−5^ quantum information processing,^6^ and quantum communication.^7−9^ In quantum sensing, the optically addressable electron spin of the nitrogen vacancy (NV) center has been successfully employed to sense various physical observables, including magnetic fields,^1^ electric fields,^2^ and temperature.^3^ In particular, scanning probe magnetometry based on single NV centers offers quantitative imaging with nanoscale resolution that enabled insights into physical systems that are inaccessible to classical approaches.^1,10^ However, the deployment of NV magnetometry in extreme conditions, such as mK temperatures and tesla-range magnetic fields, is hampered by the near-surface NV’s charge instability in cryogenic environments and limitations in coherent driving of its electronic spin, which requires driving fields of tens of GHz in frequency. Yet, nanoscale sensing under such conditions would offer exciting opportunities to address interesting condensed matter systems, such as fractional quantum Hall effects,^11^ or unconventional superconductors,^12^ by direct, nanoscale DC magnetic imaging.

The negatively charged silicon vacancy center (SiV^–^) is an alternative diamond color center hosting an electronic spin, exhibiting comparable *T*_2_^*^ to the NV center at cryogenic temperatures,^13−15^ which offers promising and advantageous prospects for single-spin quantum sensing under extreme conditions. Compared to the NV center, the SiV^–^ predominantly emits photons in the zero phonon line (ZPL) and thereby presents a more efficient spin-photon interface.^16^ Moreover, the SiV^–^ orbital and spin level structure generally allows for all-optical coherent driving of its ground-state spin.^17−19^

The inversion symmetry of SiV^–^ leads to a vanishing electric dipole moment and renders its optical transition frequency insensitive to electric field fluctuations to first order.^20^ As a result, highly coherent photon emission with linewidths limited by the inverse excited state lifetime (∼1.7 ns) have been observed for SiV^–^ centers far from the diamond surface,^21^ or even in diamond nanocrystals.^22^ Together with the SiV^–^ center’s substantial electronic spin coherence times at mK temperatures,^13^ these properties open the exciting perspective to perform all-optical, coherent nanoscale quantum sensing with SiV^–^ spins. Realizing this potential requires the ability to create SiV^–^ centers with high optical coherence within a few tens of nanometers from the diamond surface in nanostructures suited for efficient sensing operation.^23^ However, this achievement has remained elusive so far, largely because shallow SiV^–^ suffer from significant spectral instability induced by electric field noise originating from nearby diamond surfaces,^24,25^ which is further exacerbated by diamond nanofabrication. Furthermore, resonant excitation of SiV^–^, essential for all-optical sensing schemes, usually requires off-resonant charge-resetting laser pulses,^24,26−28^ that lead to additional spectral diffusion^29^ and laser heating, both of which form further obstacles to the use of SiV^–^ for quantum sensing. Here, we present a reproducible approach to address these challenges and to realize shallow (≲ 50 nm deep), single SiV^–^ centers with high optical coherence in diamond nanopillars shaped into parabolic reflectors (PRs).^23^ Our approach to SiV^–^ creation and diamond nanofabrication produces a ∼30% yield in creating close to lifetime-limited SiV^–^ centers.

Importantly, we additionally introduce an easy-to-realize charge stabilization procedure that enables such narrow linewidths in close to 100% of the shallow SiV^–^ centers in our PRs. This charge stabilization consists of a single, prolonged exposure of SiV^–^ to 445 nm laser light, which has the striking effect of narrowing the transition linewidths for SiV^–^ exhibiting initially broad lines. 445 nm laser illumination furthermore enables continuous photoluminescence excitation (PLE) measurements without any need for optical charge repumping–a PLE scheme that we refer to as charge-repump-free PLE (crf-PLE) and which we discuss further below. Most SiV^–^ we investigated after this procedure show inhomogeneous linewidths that fall within an approximate factor of 2 of the lifetime limit, with several instances showing near-lifetime-limited single sweep linewidths. In Figure 1(e), we present the narrowest transition linewidth achieved with our approach, which shows a full width at half-maximum (FWHM) Lorentzian linewidth of Δν = 100.4 ± 6.9 MHz.

**Figure 1:**
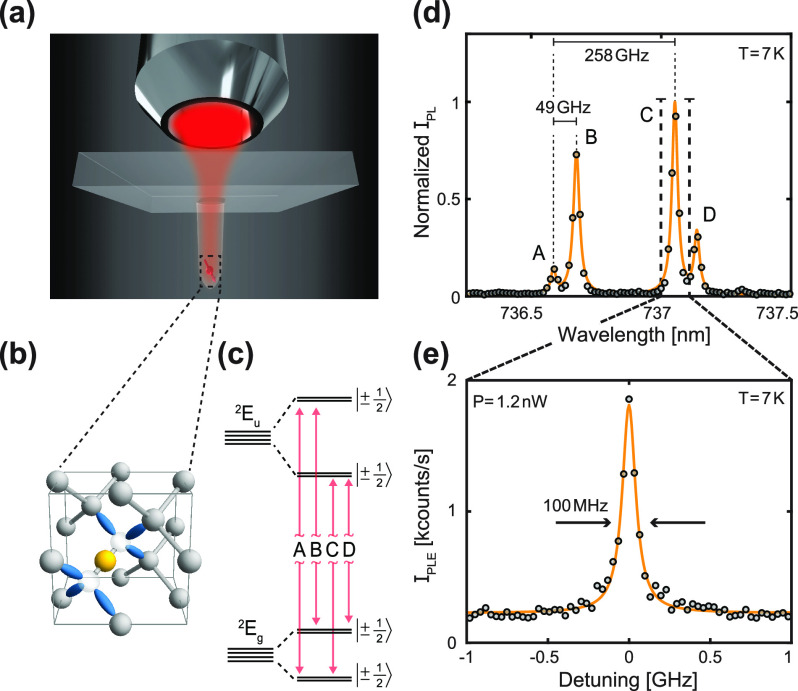
Optically coherent SiV^–^ in nanostructured diamond. (a) Rendering of the sample geometry, with an emitter placed at the focus of an overhanging parabolic reflector (PR), and optical addressing performed through the diamond substrate. (b) Atomic structure of SiV^–^, with the color center symmetry axis being oriented along the diamond 111-axis. The yellow Si atom is located at the interstitial site between two C vacancies (transparent). Nearest-neighbor C dangling bonds shown in blue. (c) Zero-field energy level diagram of SiV^–^. Red arrows denote the four zero-field optical transitions labeled A, B, C and D. (d) Typical optical spectrum of a single SiV^–^ center obtained with off-resonant laser excitation (wavelength λ = 515 nm) at 7 K, showing the zero-field optical transitions as well as the ground and excited state splittings of ∼49 GHz and ∼258 GHz, respectively. Data are shown as black dots and a four-peak Lorentzian fit in yellow. (e) Charge repump-free photoluminescence excitation (crf-PLE) measurement with 1.2 nW resonant laser power sweeping across transition C while recording the phonon sideband (PSB) intensity. Data were acquired by 14 successive laser frequency sweeps over 6 min. The Lorentzian fit (yellow) to the data (black dots) reveals a lifetime-limited linewidth of 100.4 ± 6.9 MHz.

Our diamond preparation and nanofabrication procedure is outlined in Figure 2(a): We begin with a commercially available electronic grade diamond (Element Six), sample A, which we implant (CuttingEdge Ions) with ^29^Si^+^ ions at an angle of 7°, a dose of 6 × 10^9^ ions/cm^2^, and an implantation energy of 80 keV. This energy yields a Stopping Range of Ions in Matter (SRIM) predicted emitter depth of ∼50 nm [Supporting Information (SI) Figure S1]. In order to form SiV^–^, we anneal the implanted diamond in a home-built high-vacuum oven at 400, 800 and 1300 °C for 4, 11, and 2 h, respectively. This corresponds to a slight modification of the procedure introduced by Evans et al.,^24^ where we increase the temperature of the last annealing step, as it has been shown that higher temperatures benefit the optical coherence of SiV^–^.^27^ Successful SiV^–^ creation is confirmed by observing its room-temperature (RT) ZPL around 738 nm under off-resonant optical excitation at a wavelength of λ = 515 nm. Subsequently, in order to enhance the emitters’ collection and excitation efficiencies, we nanofabricated parabolic reflectors (PRs) with diameters at the apex of ∼300 nm on the sample. For this, we use electron-beam lithography defined SiO_*x*_ etch masks and a sequence of plasma etching steps (detailed elsewhere^23^). PRs are arranged in arrays [Figure 2(b)] to facilitate both the fabrication procedure and the systematic characterization of SiV^–^. The fabricated PRs employ the same design otherwise used for diamond scanning tips in scanning NV magnetometry,^30^ which will expedite future use of SiV^–^ for scanning probe microscopy.

**Figure 2:**
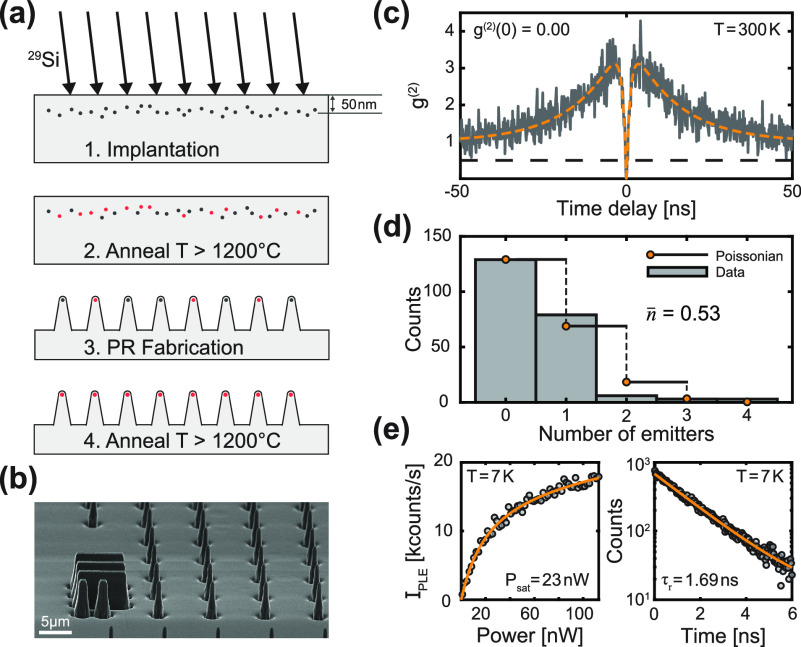
Sample fabrication and optical properties of SiV^–^ in parabolic reflectors (PRs). (a) Sample preparation workflow. First, we implant the diamond with ^29^Si^+^ at a dose of 6 × 10^9^ ions/cm^2^ at an angle of 7° and an energy of 80 keV. Then we anneal the diamond in a home-built high vacuum oven to produce SiV^–^. Third, we nanofabricate parabolic reflectors and subsequently repeat the annealing procedure from step 2 to further increase the yield of SiV^–^ formation and to enhance optical coherence.^24^ Gray dots are Si ions while red dots indicate successfully formed SiV^–^. (b) SEM image of a PR array (imaging angle 70°). Binary bulk markers on the sample are visible to the left. (c) Example of a room-temperature (RT) background corrected off-resonant *g*^(2)^(τ) recorded on a PR by exciting the emitter with a 515 nm diode laser and recording the ZPL photoluminescence (PL) intensity. The fit (dashed yellow) to the data (gray) reveals *g*^(2)^(0) = 0.00 ± 0.16, indicating a single emitter. (d) SiV^–^ number distribution and a corresponding Poissonian fit with mean n̅ = 0.53 emitters per pillar. (e) Left panel: low-temperature (LT) resonant saturation curve on transition C recorded by tuning a diode laser into resonance and varying its optical power using an AOM while collecting PSB photons. Saturation power for this particular SiV^–^ is 23.0 ± 3.1 nW. Right panel: LT ZPL PL decay of the SiV^–^ recorded with off-resonant pulsed excitation at 515 nm. Fitting (yellow line) reveals a typical optical lifetime of 1.69 ± 0.04 ns.

After diamond nanofabrication, we perform a second anneal identical to the first one, as Evans et al.^24^ have shown that the optical coherence of SiV^–^ increases by removing subsurface damage from the diamond lattice by annealing and subsequent acid cleaning. Additionally, we observe a significant increase of SiV^–^ yield after the second annealing step, as many PRs do not show a SiV^–^ ZPL immediately after fabrication at the implantation dose that we employed. Thus, the annealing steps before and after PR fabrication are a crucial ingredient for creating individual and coherent SiV^–^ centers in nanostructures.

To characterize our PR arrays, we perform systematic measurements at RT using an automized, home-built confocal microscopy setup (c.f. SI Sec. III). We measure optical spectra, off-resonant saturation curves [SIFigure S2] and off-resonant second order correlation functions *g*^(2)^(τ) for each pillar in the array. A representative data set of a background corrected *g*^(2)^(τ) recorded from a PR is shown in Figure 2(c) with a fit revealing *g*^(2)^(0) = 0.00 ± 0.16, indicating the presence of a single emitter in the PR. For background correction, we subtract from the raw autocorrelation data the uncorrelated background signal stemming from background photons, whose intensity we determined by recording photoluminescence saturation curves [see SIFigure S2]—a procedure proposed earlier by Brouri et al.^31^ [c.f. SI Sec. IV]. Subsequently, we estimate the number of emitters in a PR using the relationship , where *n* is the number of emitters,^31^ while in the absence of a SiV^–^ ZPL, we assign *n* = 0 to the PR. Using such data collected over 220 PRs, we produce a SiV^–^ number distribution, which closely follows a Poisson distribution with a mean n̅ = 0.53 emitters per pillar [Figure 2(d)]. A certain discrepancy between the data and the Poissonian fit can be assigned to uncertainties in the experimental determination of the background signal.

In the following, we present a detailed characterization of the optical properties of individual SiV^–^ under cryogenic conditions. We employ a closed-cycle coldfinger cryostat to cool the diamond sample to ∼7 K, where we conduct photoluminescence excitation (PLE) experiments. For this, we tune a narrow-linewidth diode laser near resonance with the C transition of the SiV^–^ [Figure 1(d)] and collect phonon sideband (PSB) emission as a function of excitation laser frequency.

In Figure 1(e), we present the PLE spectrum of the narrowest linewidth that we observed and that exhibits a Lorentzian FWHM of Δν = 100.4 ± 6.9 MHz. These data were obtained by averaging PLE spectra of 14 successive laser sweeps across the C transition, followed by a Lorentzian fit (for details, see next section). To benchmark the linewidth, we measure the optical lifetime of this SiV^–^ by pulsed laser excitation at a wavelength of 515 nm, followed by time-tagged ZPL photon collection [Figure 2(e), right panel]. An exponential fit to the photon decay trace yields a radiative lifetime τ_*r*_ = 1.69 ± 0.04 ns that corresponds to a lifetime-limited optical linewidth of  MHz.

To our knowledge, this is the first record of a lifetime-limited linewidth reported for ≲50 nm shallow SiV^–^ embedded in a diamond nanostructure, as required for nanoscale quantum sensing. In addition, we find resonant saturation powers of this SiV^–^ of *P*_sat_ = 23.0 ± 3.1 nW and a saturation count rate of 9.7 ± 0.7 kcounts/s [Figure 2(e), left panel], typical for our devices.

PLE experiments with solid state emitters often require regular application of optical charge-resetting pulses using off-resonant laser light.^24,26,27,32^ Such charge resetting pulses are usually applied at wavelengths between 510 to 532 nm to undo deionization events the emitter can undergo under resonant excitation. Importantly, such repumping perturbs the charge environment of the emitter and thus induces inhomogeneous broadening,^24,28,33^ precluding the observation of lifetime-limited optical linewidths. We refer to this measurement scheme as charge-repumped PLE (cr-PLE). Recently, Görlitz et al.^34^ have shown that exposing SnV^–^ centers in diamond to 445 nm laser light enables crf-PLE, reduces spectral diffusion and increases the brightness of SnV^–^. Their charge-state lifetime (i.e., effective measurement time in crf-PLE) is, however, limited to about an hour under resonant excitation. They suggest that the same approach could also be beneficial to other group-IV vacancies, such as SiV^–^.

Our experiments on charge stabilization of SiV^–^ with blue illumination revealed a similar, long-lasting effect. Prolonged (>1 h) and high-intensity (>5 mW) illumination of a PR with a 445 nm laser led to persistently bright and stable PLE emission with narrow linewidths in crf-PLE, completely removing the need of charge resetting laser pulses, which is usually necessary for our samples. A representative crf-PLE data set is depicted in Figure 3(a), where the bottom (top) panel shows a sequence of 500 single crf-PLE sweeps (c.f. SI Sec. VI) and the corresponding average, respectively. The data were continuously recorded over 14 h using exclusively near-resonant laser excitation. The PLE resonance retains its brightness and stability over the whole measurement duration and yields an averaged, inhomogeneously broadened linewidth of Δν = 211.5 ± 0.5 MHz, within a factor of 2.15 of its lifetime limit of 98.9 ± 0.9 MHz, which we evaluated by an independent excited state lifetime measurement at 7 K.

**Figure 3:**
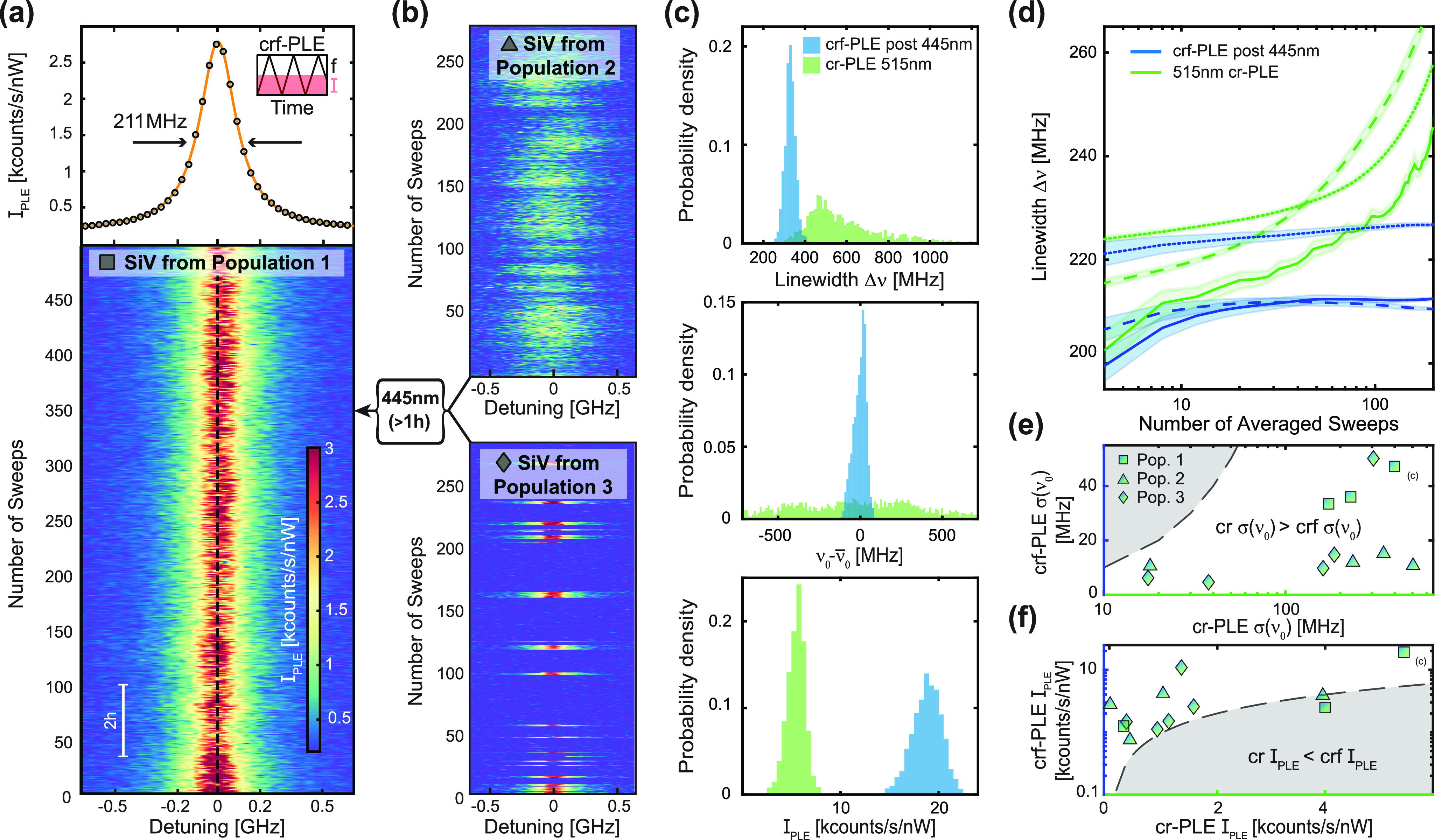
Repump-free photoluminescence excitation (crf-PLE) of SiV^–^ in diamond parabolic reflectors and charge-state stabilization with 445 nm laser light. (a) Top: crf-PLE measurement averaged over 500 single sweeps across the resonance while collecting (PSB) counts. A Lorentzian fit (yellow) to the data reports an inhomogeneously broadened linewidth of 211.5 ± 0.5 MHz, within a factor of 2.15 of the lifetime limit (98.9 ± 0.9 MHz) for this particular emitter. The inset illustrates how we conduct crf-PLE experiments with constant laser intensity while modulating the laser frequency. Bottom: Trace of the PLE measurement, showing 500 single sweeps. A dashed black line acts as a guide to the eye for zero detuning. (b) Diagram illustrating the additional populations of SiV^–^, classified by their behavior in crf-PLE (see main text). Populations 2 and 3 can be stabilized by applying high-powered (>5 mW) 445 nm laser light for extended durations (>1 h). (c) Top panel: Histogram of single sweep Lorentzian linewidths Δν of an SiV^–^ initially in population 3, which was mapped to population 1. Green bars are results from 515 nm charge-repump PLE (cr-PLE) and blue from crf-PLE after the SiV^–^ is exposed to the 445 nm laser. This representative data set shows that 445 nm exposure decreases single sweep linewidths compared to 515 nm cr-PLE. Middle panel: Center frequency spread  extracted from the same PLE measurements, revealing that line stability is dramatically improved in crf-PLE. Bottom panel: Excitation power normalized peak intensity I_PLE_ comparison for 515 nm cr-PLE and crf-PLE. (d) Lorentzian fitted PLE linewidths Δν as a function of the number of sweeps over which the PLE spectrum is averaged for three representative SiV^–^, comparing standard 515 nm cr-PLE (in green) and crf-PLE after 445 nm (in blue) illumination, exemplifying the distinct behavior between the measurement schemes. While crf-PLE converges to an average linewidth, linewidths measured during cr-PLE diverge [c.f. SIFigure S8 for more data sets]. Data sets corresponding to the same SiV^–^ are drawn with the same linestyle. The shaded area signifies the standard error. (e) Center frequency deviation σ(ν_0_) comparison between crf-PLE on the *y*-axis and cr-PLE on the *x*-axis. The data point corresponding to the histograms in (c) is marked. A dashed black line denotes y = x. Markers denote the three populations introduced in (a) and (b). (f) Comparison between 515 nm cr- and crf-PLE excitation power normalized peak intensity for the three populations as in (e), with the dashed black line denoting y = x.

Having observed the positive impact of 445 nm illumination on the optical properties of SiV^–^ in resonant excitation, resulting in crf-PLE with improved linewidths and stability, we further address the reproducibility and effectiveness of this phenomenon. To do so, we investigated 12 PRs, nine of which contain single SiV^–^, with the following measurement sequence: For each SiV^–^, and before exposing them to anything other than 515 nm laser light, we start by performing crf-PLE to assess its initial charge stability and linewidth. During these measurements, we have observed three clearly distinct, roughly equally distributed SiV^–^ populations, classified by their behavior in crf-PLE: SiV^–^ in population 1 exhibit continuous, stable and bright emission with narrow linewidths [Figure 3(a)]; SiV^–^ in population 2 present dimmer emission with large spectral diffusion and broader single sweep linewidths under continuous resonant excitation [Figure 3(b) - top]; SiV^–^ in population 3 show charge state instabilities (blinking), where it is not possible to perform continuous crf-PLE [Figure 3(b) - bottom]. Second, we perform “traditional” cr-PLE using a 515 nm charge repump laser to benchmark the PLE linewidth under this measurement scheme. Lastly, we continuously expose the SiV to 445 nm excitation at >5 mW for prolonged periods of time (>1 h), and repeat the crf-PLE linewidth measurement. From this measurement series, we found above all that SiV^–^ initially in populations 2 and 3 can be mapped to population 1 after prolonged 445 nm laser exposure, leading to drastically improved properties.

A representative result of the outcome of the above-mentioned measurement series performed on a single SiV^–^ initially from population 3 is presented in Figure 3(c). The histograms show the probability of single sweep Lorentzian linewidths Δν (top panel), center frequency spread  (middle panel) and excitation power normalized peak intensity I_PLE_ (bottom panel) for cr-PLE and crf-PLE. The data clearly show how crf-PLE after 445 nm laser exposure yields both a strongly reduced single sweep linewidth and center frequency spread, and a highly increased peak intensity compared to 515 nm cr-PLE. The results we obtained in this way for the 12 investigated SiV^–^ are summarized in Figure 3(d)–(f). In 3(d), we show three representative data sets of PLE linewidths measured on single SiV^–^ as a function of the number of single sweeps over which the data were averaged. We compare the results for 515 nm cr-PLE (green) and crf-PLE after illuminating their respective PRs with the 445 laser (blue) [see SI Section VI for more data sets]. While after a few tens of sweeps, crf-PLE converges to a linewidth in the range of ∼200 MHz, the averaged cr-PLE linewidths diverge as a function of the number of averaged sweeps. These data are testament to the absence of excess spectral diffusion, i.e. spectral wandering^35^ is completely eliminated when performing PLE without a charge repump laser, which enables long-time measurements without loss of optical coherence. Moreover, in order to further compare the two protocols, for each SiV^–^ we extracted the center frequency standard deviation σ(ν_0_) and the average power normalized peak intensity I_PLE_ from all single sweep crf-PLE spectra, and plot them against the corresponding values for the same SiV^–^ under 515 nm cr-PLE [Figure 3(e), (f)]. Lastly, we obtain a mean value of the single-sweep-averaged crf-PLE linewidths for all SiV^–^ of 223.1 ± 46.1 MHz. We consistently find that in crf-PLE, SiV^–^ exhibit higher peak PLE intensities and less background fluorescence and display strikingly reduced linewidths and spectral diffusion. However, it is noteworthy that for some SiV^–^ initially in population 1, illumination with 445 nm light slightly decreases their brightness and stability [SI Sec. VI]. We confirmed that this beneficial effect of prolonged 445 nm laser illumination is persistent and neither specific to a diamond sample nor the Si implantation dose. Specifically, we verified the same behavior as presented in Figure 3 for SiV^–^ in a second sample B, which was prepared in the same way as the first sample, but with a ^28^Si implantation dose five times higher (as a sole, slight difference between the two, we found that in the second sample, population 1 made up for a smaller percentage than in the low-dose sample A). For both samples, the beneficial effect of 445 nm laser exposure persisted throughout the time scale of this study (several months) and was neither affected by continuous, high-power laser illumination (be it resonant or off-resonant), by long idle times in the dark, nor by thermal cycling of the samples. These combined findings suggest that illumination of 445 nm laser light is a generally applicable means for permanently stabilizing the charge-environment of shallow SiV^–^ centers such that coherent optical addressing can be performed without any further charge repumping.

Only in the case where such continuous crf-PLE measurements can be conducted on a given SiV^–^ from the beginning (population 1), 445 nm laser illumination might slightly deteriorate the SiV^–^ optical properties and calls for the cautious use of blue laser illumination.

The narrow and stable SiV^–^ linewidths we demonstrated enable the addressing of individual emitters in nanostructures that contain multiple, spectrally distinct SiV^–^. Such a situation is illustrated in Figure 4(a), where a crf-PLE measurement after prolonged 445 nm laser illumination shows three PLE resonances, which we attribute to the C transitions of three separate SiV^–^ hosted in a single PR on the high-density diamond sample B. The shift in their transition frequency likely results from local variations in strain or electric field in the surroundings of each SiV^–^. A *g*^(2)^(τ) measurement conducted under off-resonant optical excitation at 515 nm [Figure 4(b)] reveals a value *g*^(2)^(0) = 0.82 ± 0.01, indicating that more than one emitter is present in this particular PR. Since, however, owing to our charge stabilization protocol, the resonances of the three SiV^–^ remain spectrally distinct, one can individually address each SiV^–^ despite their localization in a nanoscale volume. We demonstrate this by resonantly driving each of the three SiV^–^ and recording a corresponding *g*^(2)^(τ) trace. Indeed, the three photon autocorrelation traces [Figure 4(c)] all show values of *g*^(2)^(0) close to zero (*g*^(2)^(0) = 0.00 ± 0.03, 0.00 ± 0.03, and 0.00 ± 0.03, respectively), indicating that only one SiV^–^ at a time is being optically excited in this case. For nanoscale quantum sensing with SiV^–^, this result brings the interesting perspective of performing single-spin sensing in nanostructures containing small ensembles of spins, which would find immediate applications, for example, in covariance magnetometry.^36^

**Figure 4:**
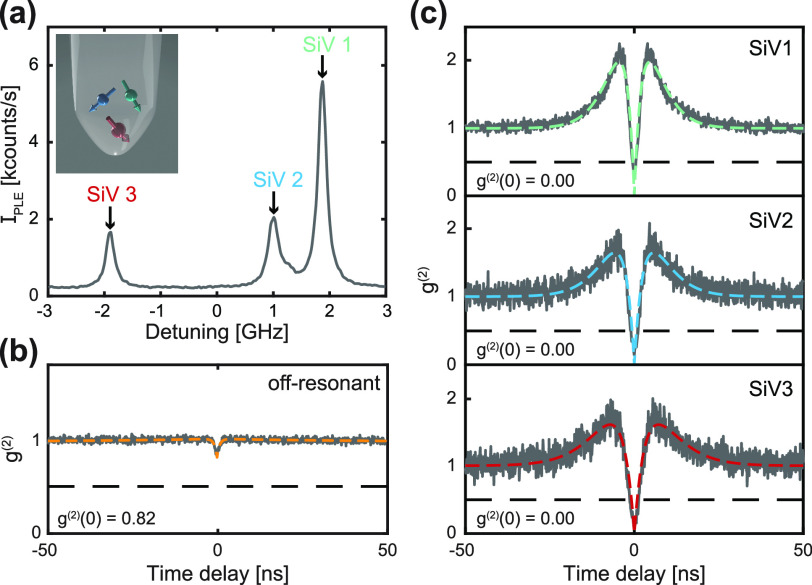
Addressing spectrally distinct individual SiV^–^ hosted in the same nanostructure. (a) crf-PLE measurement revealing three resonances, which we attribute to C transitions of three distinct SiV^–^ within the same parabolic reflector (PR). The inset illustrates such a situation. (b) Off-resonant *g*^(2)^(τ) on the PR in question, showing that indeed, more than one emitter is being addressed. (c) Resonant *g*^(2)^(τ) of each individual resonance shown in (a) obtained by subsequently tuning the 737 nm laser into each resonance and collecting (PSB) photons. These data show the ability to address one individual emitter in a multi-emitter nanostructure, increasing the yield of scanning probes in a sample.

In this work, we demonstrated the robust and reproducible creation of single narrow-linewidth SiV^–^ color centers located within a few tens of nanometers from the end facets of individual diamond nanopillars.

Nearly all SiV^–^ investigated here display inhomogeneously broadened linewidths within a factor of 2 from the lifetime limit in crf-PLE over long time scales, and single sweep linewidths that, at times, approach their respective lifetime limit. These results are enabled by a combination of a high temperature vacuum annealing step introduced after nanopillar fabrication and the application of a novel, optical charge stabilization protocol based on extended, single-time exposure of SiV^–^ to continuous-wave 445 nm laser light. The latter permanently and entirely removes the need for charge repumping in resonant excitation experiments and yields improvements in several key figures of merit of resonant optical excitation of SiV^–^ centers. Specifically, the optical charge stabilization leads to enhanced brightness, reduced spectral diffusion, and charge state preservation for those SiV^–^ which suffered from deionization under resonant excitation. While the microscopic origins underlying the demonstrated optical charge stabilization scheme remain unexplained and depletion of the charge environment may play a role,^37^ we anticipate that our results will trigger significant further research in theory and experiment.

Our results constitute a major step toward the use of SiV^–^ as nanoscale quantum sensors for applications under extreme conditions, such as single spin scanning magnetometry at sub-kelvin temperatures and tesla-range magnetic fields.^38^ Furthermore, our easy-to-implement charge-stabilization scheme will find immediate applications in other quantum technology applications of SiV^–^, including the development of quantum repeaters,^39^ quantum networks^16,40^ or indistinguishable single photon sources.^41^ Lastly, it is conceivable that our approach for generating and stabilizing near-surface color centers with high optical coherence extends to other color centers in diamond or in other wide-bandgap hosts such as hBN^42^ or SiC.^43^

## Data Availability

The data that support the findings of this study are available from the corresponding author upon reasonable request.

## Supporting Information Available

SiV^–^ implantation yield estimation, stopping range of ions in matter (SRIM) simulation results, optical characterization setup description, room temperature PR field characterization, *g*^(2)^ background correction, comparison of cr-PLE spectra with 515 and 445 nm repump pulses, PLE statistics for all data points shown in Figure 3(d)–(f), details on the PLE laser sweep, a representative power broadening measurement of a SiV^–^ in PR and a representative PLE data set of an SiV^–^ center in a third PR sample C that has not been annealed a second time (PDF)
