# Supplementary material for: Shallow Silicon Vacancy Centers with Lifetime-Limited Optical Linewidths in Diamond Nanostructures
Source: Nano Lett. 2023 Nov 21;23(23):10901–7. doi: 10.1021/acs.nanolett.3c03145 (PMC10722541; doi:10.1021/acs.nanolett.3c03145)
Supplement: Supplementary file 1 — nl3c03145_si_001.pdf [file nl3c03145_si_001.pdf]

# Supplementary Information for: Shallow Silicon Vacancy Centers with lifetime-limited optical linewidths in Diamond Nanostructures

Josh A. Zuber,[1,2,*] Minghao Li,[1,*] Marcel.li Grimau Puigibert,[1] Jodok Happacher,[1] Patrick Reiser,[1] Brendan J. Shields,[1] and Patrick Maletinsky[1,2,†]

[1]*Department of Physics, University of Basel, CH-4056 Basel, Switzerland*
[2]*Swiss Nanoscience Institute, University of Basel, CH-4056 Basel, Switzerland*
(Dated: July 24, 2023)

## I. SIV$^-$ YIELD ESTIMATION

We predicted our target implantation density of $6 \times 10^9$ ions/cm$^2$ by using yield estimates from previous samples, where we observed a Si $\rightarrow$ SiV$^-$ conversion efficiency of $\sim 0.03$ after implantation with $^{28}$Si ions at a fluence of $1.5 \times 10^{11}$ ions/cm$^2$ and 80 keV, the first annealing procedure and parabolic reflector fabrication and of $\sim 0.18$ after the second anneal.
After the first annealing and after parabolic reflector (PR) fabrication, we could still find single SiV$^-$ centers in our PRs even at this high implantation fluence, which was not the case after the second anneal. Based on simple back-of-the envelope calculations, we estimated that a fluence of $1 \times 10^{10}$ ions/cm$^2$ would yield single SiV$^-$ centers in PRs with diameters around 300 nm in combination with two annealing procedures. We then opted for the slightly lower fluence to facilitate subsequent characterization. The reason for the switch from $^{28}$Si to $^{29}$Si was based on technicalities with the implantation provider. If a single stage implanter is used, it is possible that $^{28}N_2$ is implanted alongside $^{28}$Si, leading to unwanted contamination of the sample and an increased yield of NV centers. We do not expect an impact on optical coherence based on this change.

* These authors contributed equally.
† patrick.maletinsky@unibas.ch

## II. STOPPING RANGE OF IONS IN MATTER (SRIM) SIMULATION

We estimate the depth of our emitters based on the Stopping Range of Ions in Matter (SRIM) Monte-Carlo simulations. The parameters for the simulation are as follows. For the ions, we select Si and choose a mass of 28.977 amu, an energy of 80 keV and an angle of incidence of 7 °. For the diamond target, we select C with the density of diamond of 3.53 $\mathrm{g\,cm^{-3}}$. The stopping range of the $^{29}$Si is estimated to be $54.3 \pm 13.9$ nm. Since ion channeling is not accounted for in SRIM, we expect the average depth of our emitters to be larger than this value, however, we also note that due to our two high temperature annealing steps, we expect some graphitization of the surface to occur; this graphitization layer is removed by tri-acid cleaning, bringing the emitters in our samples closer to the surface.

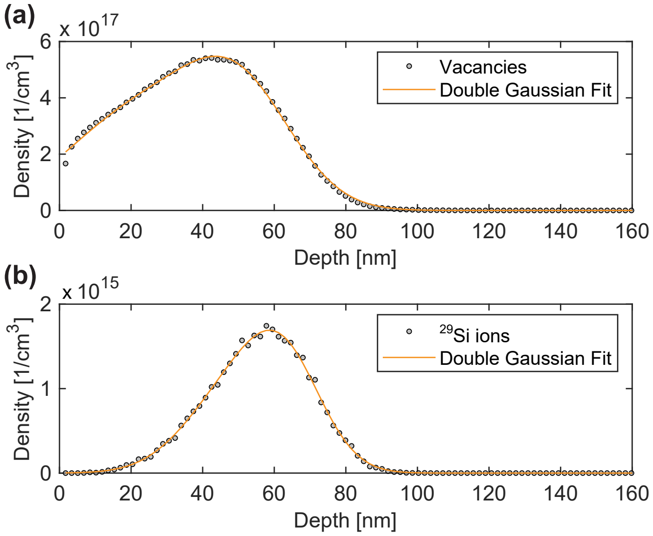


FIG. S1. SRIM results for $^{29}$Si implantation into diamond at an angle of 7 ° and 80 keV. (a) The resulting density distribution for the vacancies as a function of depth in the diamond. Fitted with double Gaussians (yellow line) in order to calculate the most likely depth of the vacancies of 44.2 nm. (b) Resulting density distribution for the $^{29}$Si ions as a function, again fitted with a double gaussian, confirming the SRIM prediction of an average depth of $54.3 \pm 13.9$ nm for the $^{29}$Si ions.

## III. OPTICAL CHARACTERIZATION SETUPS

To characterize our diamond samples at room temperature (RT), we employ a home-built confocal microscopy setup. This setup is equipped with a continuous wave (cw) 515 nm diode laser (Cobolt 06-MLD) for off-resonant excitation. At low temperature ($\sim 7$ K), the sample is housed in a variable-temperature closed-cycle cryostat (attocube attoDRY800) and we perform optical spectroscopy again with a home-built confocal microscopy setup. For this setup, we put to use cw 445 nm and cw 515 nm diode lasers (Cobolt 06-MLD) for off-resonant excitation as well as a narrow-linewidth (200 kHz) tunable (720-739 nm) diode laser (Sacher Lasertechnik LION) for resonant excitation. Frequency modulation and control of the LION is achieved through a PID loop of a HighFinesse WS-U wavelength meter. Additionally, we stabilize the intensity of the resonant laser through the PID loop of a home-built optical intensity stabilization (OIS) module (Physics Basel SP 999). In case of the resonant laser and if applicable to the experiment in question, we carve pulses with an acousto-optical modulator (AOM, G&H R15210), whereas the off-resonant lasers can be electronically modulated out of the box. For both RT and LT, we focus laser light onto the sample with a 0.8 NA objective (Olympus LMPLFLN 100X) thermalized at RT, through which we also collect the signal. Photons are detected by silicon-based single-photon counting modules (SPCM, Excelitas AQRH-33-FC). We perform photon-correlation measurements using a time-correlated single-photon counting (TCSPC) system (PicoQuant PicoHarp 300) connected to the SPCMs. For optical lifetime measurements we use a pulsed laser (NKT Photonics SuperK Extreme EXW-12) tuned to 515 nm and said TCSPC system. Optical spectra are recorded on the camera of a spectrograph (Princeton Instruments SP-2500).

## IV. ROOM TEMPERATURE PR FIELD CHARACTERIZATION

The room temperature (RT) PR characterizations shown in the main text are performed on 220 PRs investigated in two array fields (confocal scans shown in Fig. S3(a) and (c)). As the arrays are regularly fabricated, a series of systematic measurements can be easily performed on each PR. For every PR in the selected array, a ZPL spectrum measurement is conducted to filter out the empty PR that contain no detectable $SiV^-$. Then, for every non-empty PR, a saturation curve, $g^{(2)}(\tau)$ and RT excited state lifetime measurement are carried out successively. An exemplary RT ZPL spectrum under off-resonant excitation with a 515 nm laser is shown in Fig. S2 (a) with the associated saturation curve shown in its inset. Fig. S2 (b) and (c) present the histograms of the ZPL linewidths and position for non-empty PRs. These results clearly show that the $SiV^-$ centers we created in PRs are highly homogeneous and mostly unstrained. Fig. S3 (b) and (d) shows the number of SiV in the corresponding PR in the confocal scan in Fig. S3 (a) and (c) obtained by the systematic $g^{(2)}(\tau)$ measurements.

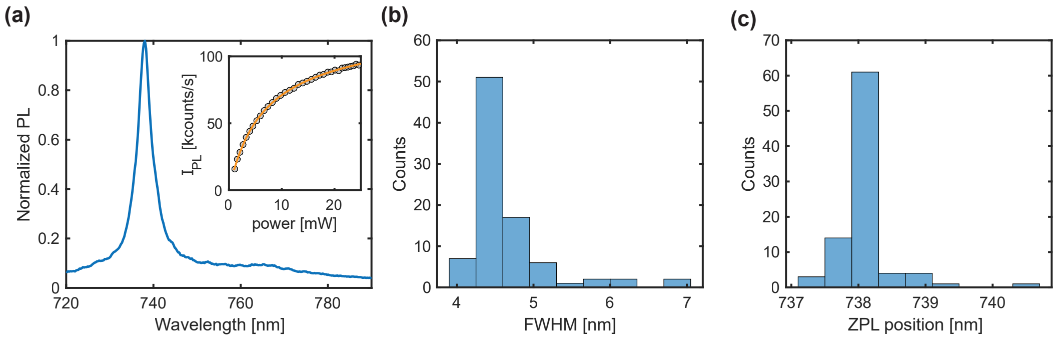


FIG. S2. (a) Example of a typical room temperature photoluminescence (PL) spectrum of the $SiV^-$ center, the zero-phonon line (ZPL) of $SiV^-$ centered at around 738 nm. The inset shows the room temperature saturation curve when collecting ZPL photons upon off-resonant excitation at 515 nm (gray scatters) with the fitting (yellow line). (b) and (c) Histograms of the linewidth (FWHM) and the ZPL position, obtained by fitting the room temperature spectra with a Lorentzian, of in total 88 pillars that contain detectable $SiV^-$ centers from sample A.

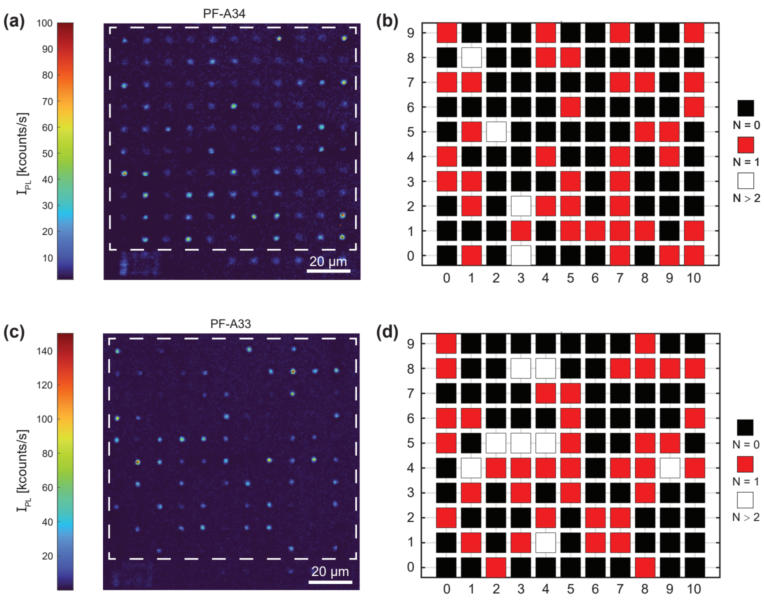


FIG. S3. (a) and (c) Confocal scans of the two PR arrays where we perform the systematic characterization (optical spectra, saturation curve, lifetime and $g^{(2)}(\tau)$ measurements as mentioned in the main text) at room temperature (PRs inside the white dashed box). (b) and (d) respectively present the result of the number of emitters embedded in the PRs at the corresponding coordinate, facilitating recognition of individual PRs when the sample is transferred to the cryostat for low temperature characterizations. The histogram presented in Fig. 2(d) in the main text is based on these results.

## V. $g^{(2)}(\tau)$ BACKGROUND CORRECTION

For the background correction of second order correlation function $g^2(\tau)$ data, we adopt the method introduced by Brouri et al. [1]. The raw data $W(\tau)$ is obtained in the histogramming mode of the PicoHarp 300 by measuring the waiting time between two photon detectors in the Hanbury Brown and Twiss (HBT) setup. Since we operate in the low count rate regime, the histogram serves as a reliable approximation of the exact photon correlation obtained through full counting statistics in time tagger mode. Therefore, we normalize the raw data $W(\tau)$ by dividing it by its value at a large time delay ($\tau \gg \tau_c$) where $W(\tau)$ tends towards a constant value.

$$g^{(2)}_{norm}(\tau) = \frac{W(\tau)}{W(\tau \gg \tau_c)} \tag{1}$$

This normalization method is validated by comparing the value of $W(\tau \gg \tau_c)$ with the coincidence number $C$ obtained from an ideal Poissonian photon source:

$$C = N_1 N_2 \Delta t T, \tag{2}$$

where $N_1, N_2$ are the count rates on detectors 1 and 2 respectively, $\Delta t$ is the selected time bin width and $T$ is the total acquisition time. In our measurement, although we do not have access to exact $N_1, N_2$ during the acquisition, the current count rate on each detector is saved every five minutes. We found that in most of the cases, $C \sim W(\tau \gg \tau_c)$.
After normalizing the raw data, we further correct for the background by the signal-to-background ratio $\rho(P) = S(P)/(S(P) + B(P))$ for a given power $P$. This value is determined individually for each SiV$^-$ center by fitting the saturation curve and extracting the values of S(P) and B(P) from the fit at the laser intensity used to acquire the corresponding $W(\tau)$. The background corrected $g^{(2)}_{\text{corr}}(\tau)$ is then given by

$$g^{(2)}_{\text{corr}}(\tau) = \frac{g^{(2)}_{\text{norm}}(\tau) - (1-\rho(P)^2)}{\rho(P)^2}. \tag{3}$$

Here, we take the $g^2(\tau)$ data shown in figure 2 (c) in the main text as an example illustrating how such correction is performed and affects the result of a $g^2(\tau)$ raw data in Fig. S4.

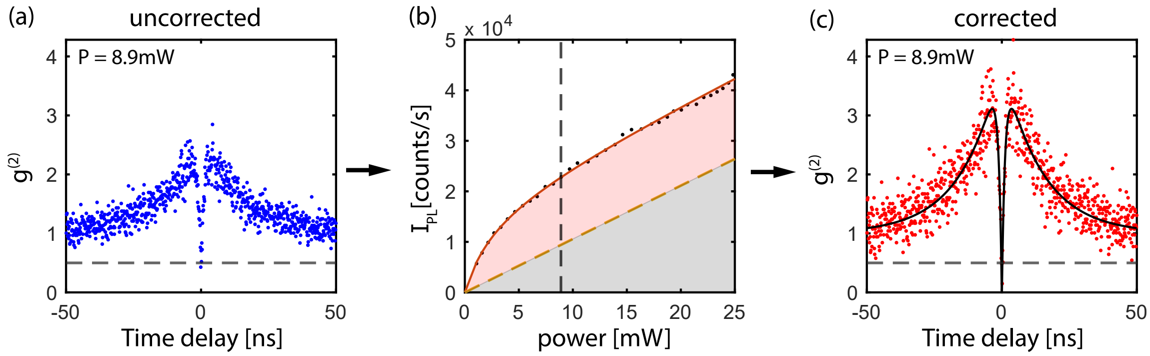


FIG. S4. (a) The uncorrected raw data of the off-resonant $g^2(\tau)$ data displayed in Fig. 2(c) of the main text. It was acquired for a ZPL collection under 515 nm excitation at 8.9 mW. (b) The saturation curve of ZPL emission under 515 nm excitation with fitting marked as the red line. From the fitting, we can distinguish the contribution of ZPL emission (pink area) and the background (gray area) for each power. The dashed vertical line denotes 8.9 mW. (c) The back ground corrected $g^{(2)}$ data.

## VI. CR-PLE WITH 515 nm AND 445 nm REPUMP PULSES

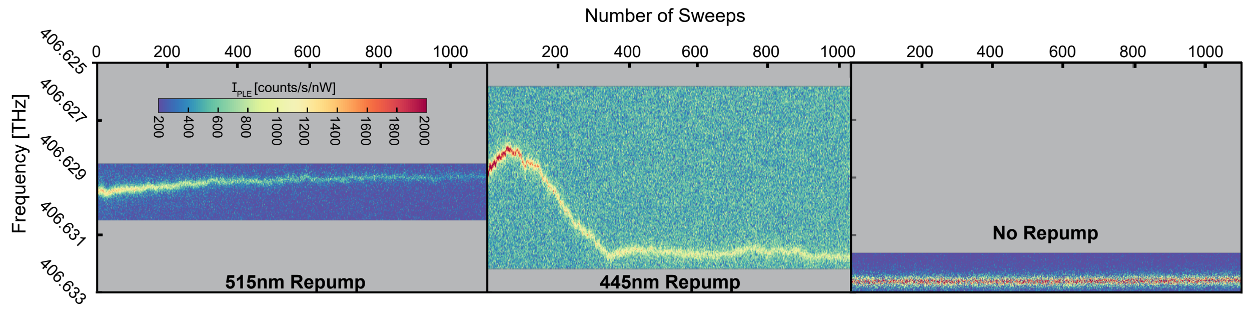


FIG. S5. PLE traces for different repump schemes on the same PR. Left panel: For 515 nm cr-PLE, we observe spectral diffusion on the order of below 1 GHz, while for 445 nm cr-PLE (middle panel), spectral diffusion first increases to around 2 GHz before saturating. Finally, in a crf-PLE (right panel), spectral diffusion is minimized, albeit the resonance shifts slightly from the saturated frequency observed in the 445 nm cr-PLE. These experiments were performed one after the other with some time in the dark between each measurement. A sweep takes around 26 s for the first and third measurement and around 64 s for the second measurement, owing to its larger range.

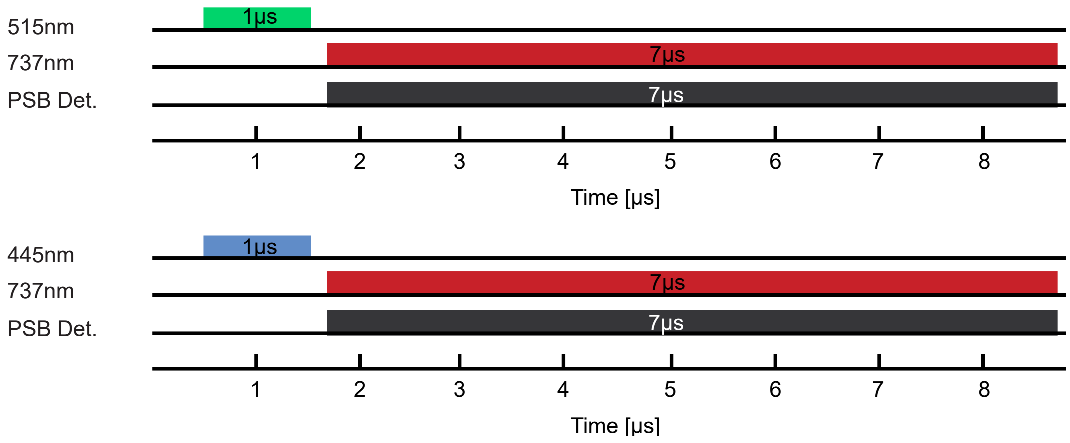


FIG. S6. Exact pulse sequences for cr-PLE measurements with 515 nm and 445 nm respectively. Off-resonant repump pulses have a length of 1 µs whereas the resonant laser pulse and the detection window have a length of 7 µs

## VII. PLE STATISTICS AND SINGLE SWEEP DISCUSSION

The statistics of PLE spectral quantities (linewidth, peak position and peak intensity) presented in the main text are extracted from so-called single sweeps along a long PLE measurement. In this section, we provide a detailed description of the actual laser sweeps in our PLE measurements and how we define the term "single sweep". The actual laser (forward-) sweep is achieved by sequentially adjusting the set point from the initial to the final detuning frequency (or vice-versa, respectively, for the backwards sweep), with each set point regulated by a PID loop via a wavemeter (HighFinesse WS-U). The dwell time for the laser at each set point is set to be $\sim 0.2$ s allowing the PID loop sufficient time to reach the set point, while PL intensity is measured by an APD with an integration time of 20 ms. The resulting back-and-forth actual laser sweeps possess a saw-tooth shape as shown in the lower panel of Fig. S7. A typical number of set points we choose is 61 points for a one-way laser sweep across a detuning range from $-1$ GHz to 1 GHz. The total time for one back-and-forth laser sweep is $\sim 26$ s. In order to strike a balance between a satisfactory signal-to-noise ratio in single sweep PLE spectra and ensuring the visibility of spectral diffusion dynamics, we define here two complete back-and-forth actual laser sweeps (or four one-way laser sweeps) to be one "single sweep" as illustrated in Fig. S7, i.e. we average the data over these four actual laser sweeps to produce what we call a "single sweep". When analyzing the statistics of PLE spectral quantities of a single sweep, we adopt the overlapping sampling scheme outlined in the lower panel of Fig. S7, where for a total number of actual laser one-way sweeps $N$, the number of samples for a single sweep would be $N-3$. However, when displaying a PLE trace as shown in upper panel of Fig. S7, the successive four one-way sweeps compose the single sweep in the trace.

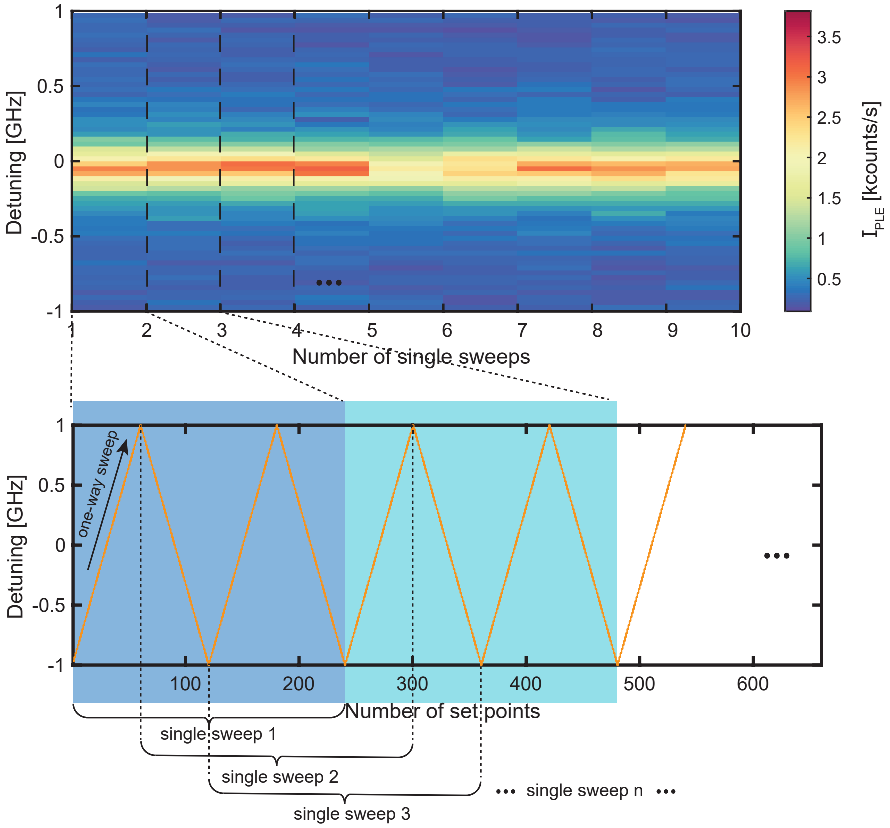


FIG. S7. The upper panel shows an exemplary PLE trace with a total number of 9 single sweeps with a sweeping range from -1 GHz to 1 GHz. One single sweep PLE spectrum in this trace consists of two entire actual back-and-forth laser sweeps from -1 GHz to 1 GHz as sketched in the lower panel. As one single sweep data set in the trace is composed of four one-way laser sweeps across the transition, the single sweeps can be sampled in an overlapping scheme as shown in the lower panel.

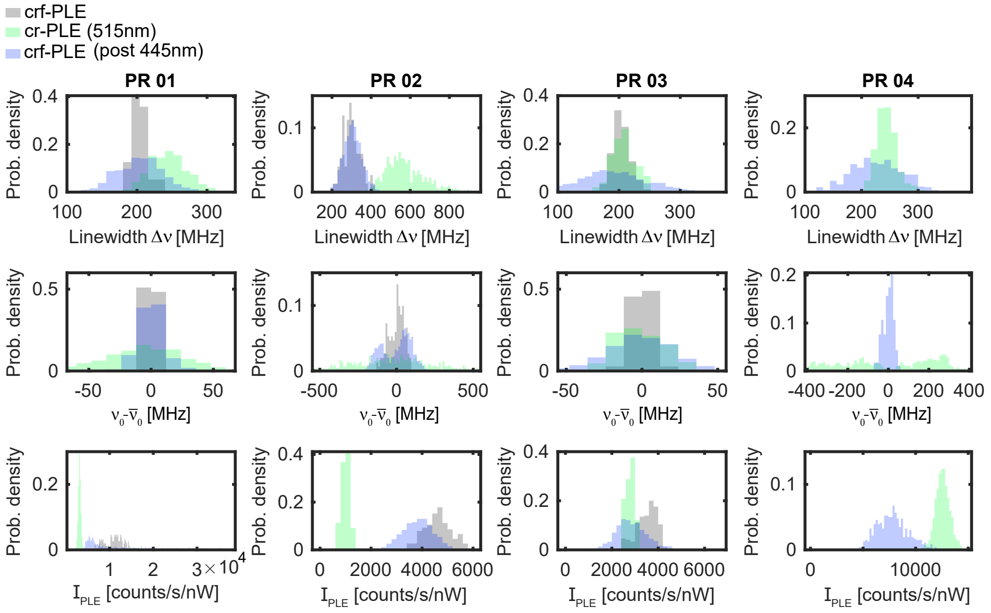
crf-PLE
cr-PLE (515nm)
crf-PLE (post 445nm)
PR 01
PR 02
PR 03
PR 04
Prob. density
Linewidth Δν [MHz]
$\nu_0$-$\bar{\nu}_0$ [MHz]
$I_{PLE}$ [counts/s/nW]

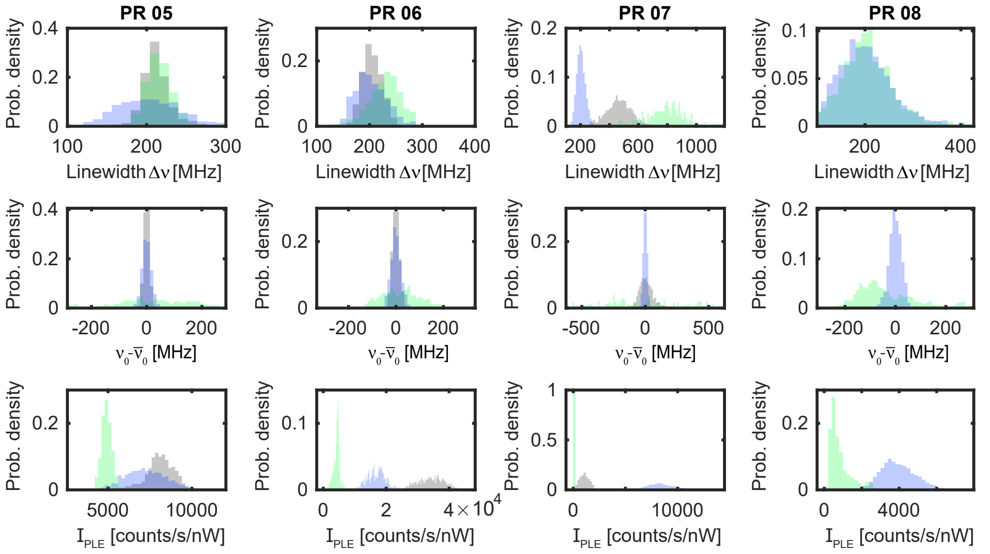
PR 05
PR 06
PR 07
PR 08
Prob. density
Linewidth Δν [MHz]
$\nu_0$-$\bar{\nu}_0$ [MHz]
$I_{PLE}$ [counts/s/nW]

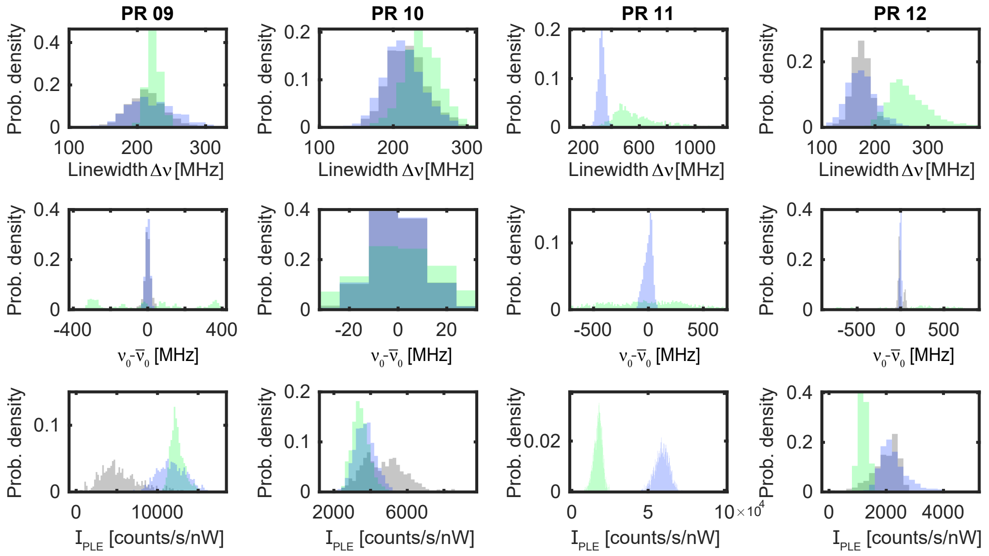


FIG. S8. Single sweep linewidths $\Delta\nu$, center frequency spread $\nu_0 - \bar{\nu}_0$ and peak intensity $I_{PLE}$ histograms for 12 PRs, comparing crf-PLE before 445 nm exposure (grey, if possible for the defect center in question), 515 nm cr-PLE (green) and crf-PLE after 445 nm exposure (blue).

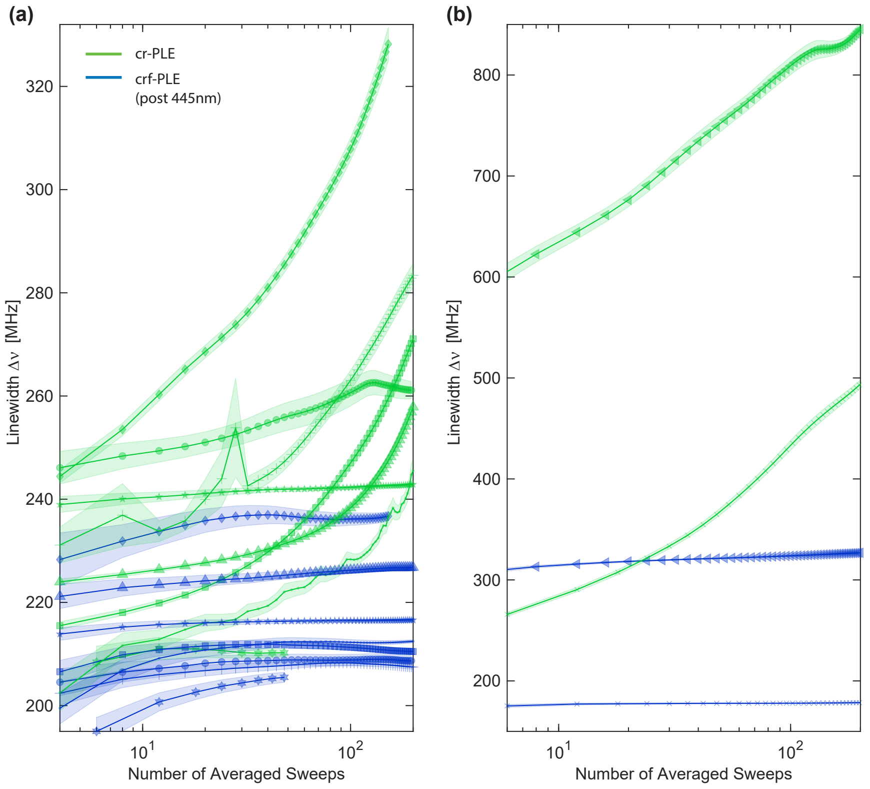


FIG. S9. Linewidth $\Delta\nu$ evolution as a function of the number of averaged sweeps, additional data sets. (a) $\Delta\nu$ evolution under crf-PLE (blue) and cr-PLE with 515 nm repump pulses (green) for PR 01, PR 03-06, PR 08-10. Symbols used correspond to the same PR. The shaded area denotes the standard error. (b) $\Delta\nu$ evolution under crf-PLE (blue) and cr-PLE with 515 nm repump pulses (green) for PR 02, PR 07. Symbols used correspond to the same PR. The shaded area denotes the standard error.

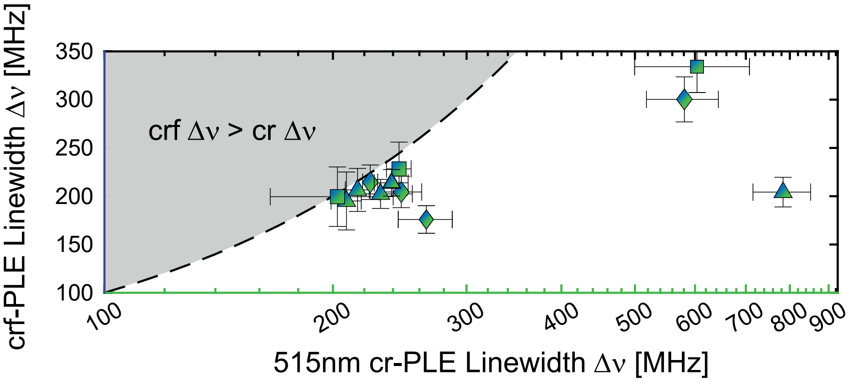


FIG. S10. Linewidth $\Delta\nu$ comparison between crf-PLE and cr-PLE including error bars. Squares, diamonds and triangles correspond to the three populations identified in the main text. Note the logarithmic scale on the x-axis. The dashed line denotes x=y.

## VIII. POWER BROADENING

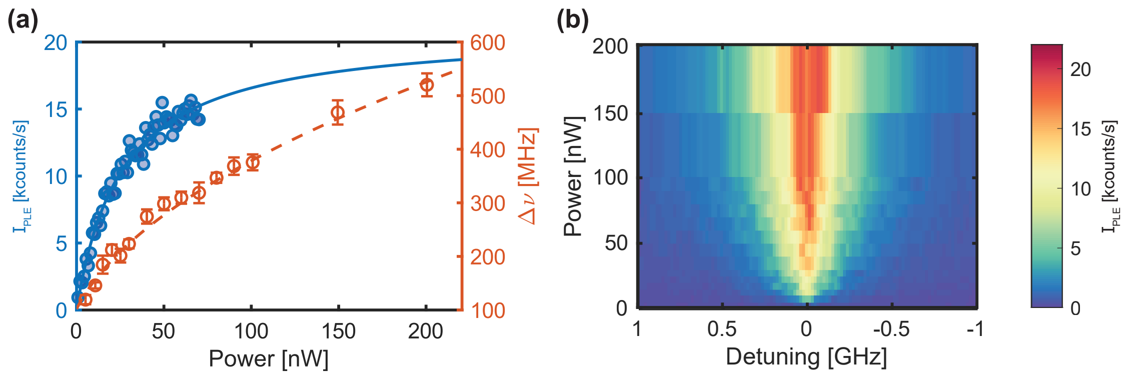


FIG. S11. Powerbroadening of an exemplary $SiV^-$. (a) shows the saturation curve measured under resonant excitation of a single $SiV^-$ in PR (blue scatter) with the fitting (blue line) superposed with the linewidth of the emitter measured with increasing excitation powers fitted with the power dependent linewidth $\Delta\nu(P) = \Delta\nu_0\sqrt{1+P/P_{sat}}$, giving the linewidth at zero power, $\Delta\nu_0 = 100.3$ MHz. (b) shows the averaged PLE measurements of this PR with different excitation powers from 1 nW to 200 nW.

## IX. PLE LINEWIDTH ON SAMPLE C

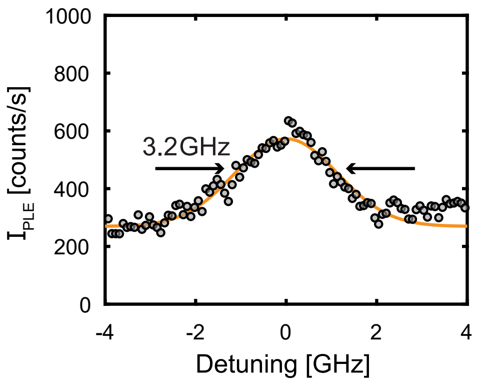


FIG. S12. crf-PLE linewidth on a third sample C with parabolic reflectors. This sample has not been annealed a second time, exhibiting solely broad linewidths on the order of GHz. This sample was implanted with $^{28}$Si ions at 25 keV.

[1] R. Brouri, A. Beveratos, J.-P. Poizat, and P. Grangier, Photon antibunching in the fluorescence of individual color centers in diamond, **25**, 1294.
